# Development of a video-based education and process change intervention to improve advance cardiopulmonary resuscitation decision-making

**DOI:** 10.1186/s12913-016-1803-x

**Published:** 2016-10-06

**Authors:** Nicholas Waldron, Claire E. Johnson, Peter Saul, Heidi Waldron, Jeffrey C. Chong, Anne-Marie Hill, Barbara Hayes

**Affiliations:** 1 Department of Rehabilitation and Aged Care, Armadale Kelmscott Memorial Hospital, 3056 Albany Highway, Armadale, 6112 Western Australia Australia; 2 Health Strategy and Networks, System Policy and Planning, Department of Health, Government of Western Australia, 189 Royal Street, East Perth, 6004 Western Australia Australia; 3 School of Medicine, University of Notre Dame, 32 Mouat St, Fremantle, 6959 Western Australia Australia; 4 Cancer and Palliative Care Research and Evaluation Unit, School of Surgery, The University of Western Australia, 35 Stirling Hwy, Nedlands, 6009 Western Australia Australia; 5 John Hunter Hospital, Lookout Rd, New Lambton Heights, Newcastle, NSW 2305 Australia; 6 Intensive Care, Newcastle Private Hospital, 14 Lookout Rd, New Lambton Heights, Newcastle, NSW 2305 Australia; 7 Clinical Teaching - Public Hospitals and Curriculum Development Communication and Clinical Practice Domain, School of Medicine, University of Notre Dame, 32 Mouat St, Fremantle, 6959 Western Australia Australia; 8 School of Physiotherapy and Exercise Science, Curtin University, Kent St, Bentley, 6102 Western Australia Australia; 9 Advance Care Planning Program, Northern Health, 85 Cooper St., Epping, VIC 3076 Australia; 10 Medical School, University of Melbourne, Parkville, VIC 3010 Australia

**Keywords:** Advance cardiopulmonary resuscitation, CPR decision-making, Goals of care, Advance care planning, Medical education

## Abstract

**Background:**

Advance cardiopulmonary resuscitation (CPR) decision-making and escalation of care discussions are variable in routine clinical practice. We aimed to explore physician barriers to advance CPR decision-making in an inpatient hospital setting and develop a pragmatic intervention to support clinicians to undertake and document routine advance care planning discussions.

**Methods:**

Two focus groups, which involved eight consultants and ten junior doctors, were conducted following a review of the current literature. A subsequent iterative consensus process developed two intervention elements: (i) an updated ‘Goals of Patient Care’ (GOPC) form and process; (ii) an education video and resources for teaching advance CPR decision-making and communication. A multidisciplinary group of health professionals and policy-makers with experience in systems development, education and research provided critical feedback.

**Results:**

Three key themes emerged from the focus groups and the literature, which identified a structure for the intervention: (i) knowing what to say; (ii) knowing how to say it; (iii) wanting to say it. The themes informed the development of a video to provide education about advance CPR decision-making framework, improving communication and contextualising relevant clinical issues. Critical feedback assisted in refining the video and further guided development and evolution of a medical GOPC approach to discussing and recording medical treatment and advance care plans.

**Conclusion:**

Through an iterative process of consultation and review, video-based education and an expanded GOPC form and approach were developed to address physician and systemic barriers to advance CPR decision-making and documentation. Implementation and evaluation across hospital settings is required to examine utility and determine effect on quality of care.

**Electronic supplementary material:**

The online version of this article (doi:10.1186/s12913-016-1803-x) contains supplementary material, which is available to authorized users.

## Background

Cardiopulmonary resuscitation (CPR) is frequently administered as the default treatment for all patients whose heart stops beating unless a withhold order exists [1]. Survival to discharge following in-hospital CPR ranges between 0 and 32 % [2]. Hence, for many patients, CPR is futile or associated with undesirable disability [3]. Not-for-resuscitation (NFR) orders have been introduced in most countries to prevent the use of CPR in situations when it is deemed futile or unwanted by patients, however, NFR orders are challenging to complete, performed infrequently or too late to seek patient preferences, and have been correlated with reduced quality of care [4]. The introduction of Rapid Response Teams (RRTs) aimed to intervene before cardiac arrest and improve outcomes. However, around one-third of RTT calls are for people at end-of-life which may not have been recognized by the treating team [5]. Deaths following RRT review remain high at 25 % [6] without improvement to end-of-life care [7].

Medical training focuses on life support, with minimal education related to communication and in-advance decision-making about CPR and treatment limitation [8]. Performing CPR when patient preferences have not been sought may constitute unwanted care [9] and the absence of clear escalation plans makes providing the best ‘in the moment’ medical decisions difficult when patients can no longer speak for themselves [10]. Although an ethical framework for advance CPR decision-making and discussion has been developed, such conversations with patients themselves are not regularly conducted [11]. In Australia, a ‘goals of care’ approach is proposed to improve in-advance decision-making (hereafter referred to as advance CPR decision-making) and documentation related to treatment limitations [12]. Such changes may improve quality of care by aligning patients’ goals of care and the treatment provided.

Hospitals are an important setting for advance care planning, with death often preceded by increasingly frequent admissions, although prognostication can be difficult with uncertain outcome for any individual admission. CPR decision-making forms part of a complete treatment plan that incorporates both appropriate escalation and limitations. This plan should also address end-of-life care issues, as well as be repeated and adapt over time as the patient condition changes [13].

We aimed to explore physician barriers to advance CPR decision-making in the hospital setting and develop a pragmatic intervention to support clinicians to undertake, and document, routine advance care planning discussions. This article describes the development of a two-pronged strategy—an education video and resources for teaching CPR decision-making, and processes to support clinicians to undertake and document, regularly conducted advance care planning discussion—building on the medical Patient Goals of Care summary and processes described by Brimblecombe et al. [14].

## Methods

**Fig. 1:**
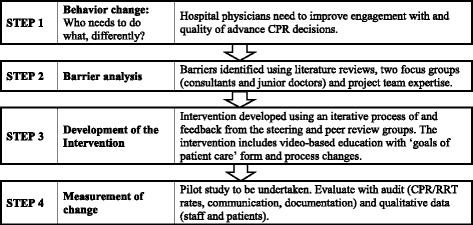
Steps to develop an intervention to improve advance CPR decision-making in the hospital setting. CPR: Cardiopulmonary resuscitation, RRT: Rapid response team calls

In developing the intervention to change practice, a four-step framework was utilized (Fig. 1) [15]. In Step 1, our desired behavior change was to improve physician advance CPR decision-making in hospital medical wards, as a surrogate of a complete care plan. This paper describes Steps 2 and 3 in detail, namely the barrier analysis and intervention development, undergoing trial in multiple Western Australian hospitals. Step 4 is evaluation of the implementation. The evaluation is currently being conducted and will be published elsewhere.

An iterative process was used to develop two intervention elements: (i) an education video and resources for teaching advance CPR decision-making and communication; (ii) an updated ‘Goals of Patient Care’ (GOPC) form and process. The GOPC used existing documentation [14] which was reviewed and amended in multiple cycles by the project steering and peer review groups (Additional file 1: Table S1). Adult educational theory was used to inform development of the teaching resources [16].

The project was conducted between April 2014 and May 2015 in a 290-bed general hospital in Perth, Western Australia (WA). It was assessed by hospital governance and approved as quality improvement. Reference # CRHDGolA27.2.

### Analysis of barriers and enablers

Barriers and enablers to advance CPR decision-making were identified through a literature review, a review of existing programs, guidelines and policies that demonstrated innovative CPR decision-making practices, and by conducting two focus group discussions. Relevant studies were identified by searching the PubMed database and through snowballing review of reference lists, and expert input from the steering and peer review groups. Data relating to barriers and enablers were extracted and compiled by authors NW and JC. Author BH negotiated and resolved any discrepant views on inclusion and exclusions.

Participants volunteered for two focus groups in response to an email invitation from author NW. Written informed consent was obtained. Focus group questions were designed to elicit: (i) thoughts and feelings about conducting CPR; (ii) experiences of advance CPR decision-making; (iii) suggestions for improvement. The first focus group comprised 10 junior doctors (interns, resident medical officers, registrars) and the second comprised eight consultants (intensive care, emergency, general and geriatric medicine).

The focus group discussions were audio-taped and transcribed, at which point all identifying information was removed. White-board notes were included for analysis. Identified barriers were informed by Cochrane et al’s systematic review of how barriers to optimal healthcare are assessed [17] and were grouped into categories and thematically analysed in conjunction with the pre-existing literature [18].

## Results

### A barrier and enabler analysis results

Three themes emerged from the qualitative analysis, identifying where improvements in advance CPR decision-making could be made: (i) knowing what to say; (ii) knowing how to say it; (iii) wanting to say it. Findings from the literature were consistent with the focus group findings. A summary of reported barriers, their source and recommended interventions is in (Table 1). Following is a discussion of key barriers, enablers and the resulting clinical approach. Detailed quotes are provided in Additional file 1: Table S2.

**Table 1:** Barriers to routine advance CPR decision-making in hospitals with recommended interventions

Themes with barrier (category)	Description	Recommended Intervention
(i) Knowing what to say		
Lack of knowledge (cognitive)	Uncertain how to optimally perform the medical assessment *(JFG)* Falsely high expectation that a useful predictive tool exists *(JFG)* Range of experiences of CPR outcomes *(JFG)* Poor understanding of differences between active and palliative management [22] *(L, JFG, CFG)*	Staff education using video resource - Outline of medical assessment process including statistics, uncertainty and how this relates to overall treatment plan. - Promote palliative care as an active treatment option. - Demonstrate frailty and different health trajectories which can trigger the use of the ‘surprise question’^a^and SPICT tool [35] in the assessment process. - Propose use of a consistent approach using ethical framework [11]. - Provide statistics for outcome in different settings and use of statistics in applying the Goals of Patient Care decision-making framework.
Lack of skill/expert clinical reasoning (cognitive)	Difficulty predicting patient trajectory and outcomes *(L, JFG, CFG)* Juniors evaluate prognosis intuitively [19] *(L, JFG)* Difficulty in coming to a decision [19] *(L, CFG)* Wide variation in approach modeled by consultants *(JFG, CFG)*	
Lack of evidence utility (guideline)	Guidelines only address technical aspects of CPR [20] *(L)* Difficult to relate CPR outcome data to individual patients *(CFG)* Potential for worse care with NFR decision [21] *(L, CFG)*	
(ii) Knowing how to say it		
Lack of self-efficacy (attitudinal)	Range of views about role the family and patient play in coming to a decision *(JFG, CFG)*	Staff education using video resource - Recommend routine engagement with scripted questions - Promote conversations are rewarding and desired by consumers. - Acknowledge the challenge of emotional distress but that communication skills can be learnt and specific strategies to deal with emotions. - Promote the benefit of discussing patient preferences with patient and family members. Goals of Patient Care Process - Supports routine use of two scripted questions by junior doctors to attain surrogate decision-makers and advance care planning - Normalise conversations as routine care, with decisions viewed as part of overall treatment plan. - Promote consultants to refine skills, lead and mentor communication skills. - Promotes the doctor as a medical expert using a shared decision-making approach
Lack of confidence in ability (emotive)	Juniors experience discomfort or embarrassment [19, 28] *(L, JFG, CFG)* Concerns regarding potentially offending patients and may upset them *(L, JFG, CFG)* A desire not to cause anxiety or distress [28] *(L)*	
Lack of knowledge about patient (cognitive)	Difficult discussing resuscitation with patients whom they did not know [28].(*L, JFG, CFG)*	
Lack of knowledge (cognitive)	Juniors feeling unskilled to undertake task [33] *(L,JFG)*	
Lack of peer guidance and role models (physician)	Poor training for decision making and communication [32, 33] *(L, JFG, CFG)* Lack of modeling and mentoring by consultants *(JFG)*	
Conflicting culture (patient)	Patients have falsely high expectations of CPR outcome *(L, JFG, CFG)* Discrepancy between patient and family desire for CPR *(JFG, CFG)*	
(iii) Wanting to say it		
Awareness (cognitive)	Under-estimate patients wanting discussion [26, 28] *(L)* Families can be unaware of the terminal status of patient [27] *(L, JFG)*	Staff education using video resource - Acknowledge that doctors are the main barriers with patients willing to engage. - Appreciate that the area is new and consultant also required to improve skills. - Acknowledge that all doctors have a role to engage in discussions and collaborate with collegues. Goals of Patient Care Process - Outline clear roles for junior and senior staff. - Audit rates of decisions, decision-making process and communication levels. - Provide organizational endorsement. - Allow clinicians to undertake discussions in practical manner and build capacity, without imposing mandated targets. - System changes to routinely seek patient preferences - View limitations to escalation plans as still receiving active care by describing as a goal of care. - Update policy in line with improved clinical care. - Emphasis the benefits by the process extending beyond current admission.
Lack of accurate self-assessment (attitudinal)	Perceive problems with other practitioners, not themselves [23] *(L, JFG, CFG)* Juniors over emphasise abilities [28] *(L, JFG, CFG)* Poor insight into substandard communication [23] *(L, JFG, CFG)*	
Lack of sense of authority (emotive)	Juniors feel don’t have decision-making authority, they feel disempowered and frustrated *(JFG)*	
Lack of motivation (physician)	Consultants express frustration at inaction of others *(CFG)* Consultant inertia, poor ownership and avoidance *(CFG)*	
Legal concerns (physician)	Fear of complaint [31] *(L, JFG, CFG)*	
Time and support (resource)	Time pressures to complete rounds *(JFG, CFG)* Inadequate time to establish rapport with patient *(JFG, CFG)* Difficult to set aside time and co-ordinate meetings *(JFG, CFG)*	
Workload/overload (system)	Competing demands with CPR decisions dropping in priority *(CFG)*	
Organizational (process)	Variable triggers to have a discussion with range of views on when to have conversation [34] *(L, JFG, CFG)*	
Lack of harmony (system)	Policies out of date with contemporary practice *(CFG)*	

*JFG* Junior focus group; *CFG* Consultant focus group; *L* Literature

^a^Surprise question: Would you be surprised if this patient died within the next 12 months?

#### Knowing what to say

The clinical aspects of completing a medical assessment to inform a discussion with patients about their resuscitation plan was well understood by doctors in both focus groups. Both consultants and junior doctors identified the patient’s illness trajectory, current problems and potential reversibility, together with an assessment of the patient’s likelihood of having a cardiac arrest, reversibility of that arrest and the possible outcomes if treated with CPR as integral to such discussions. However, focus group participants identified lack of knowledge and expert clinical reasoning as barriers to advance CPR discussions. Consultants reported that junior doctors had a lack of understanding of the concepts of care escalation, palliative care and advance care planning, with health services still focused on life prolonging treatment for all patients. Conversely, it was identified that there needs to be multiple levels of escalation of care because *“many things can happen that are acutely reversible”(CFG)*. Consultants reported that an emphasis on the treatments that *are appropriate* and *will be provided* facilitates difficult discussions and improves quality of care.

*Barriers for the junior staff in not discussing it [CPR] are a lack of understanding about the implications and also the different nuances of it as well, you know, completely not for CPR versus not for DC cardioversion, for intubation, for ICU, what are ceilings of care, are you still for active medical management, are you completely pulling out and palliating? (CFG)*

It was acknowledged, particularly by junior doctors, that it is quite difficult to define futile treatment for individual patients. Younger patients who are clearly in the default position (i.e. all life-sustaining treatment) and those who are clearly dying did not create clinical challenges. Participants were most challenged by the assessment of patients on medical wards and those with complex clinical presentations, multiple comorbidities and advancing age and by the very aged person who is quite well.

*I present this 70 year old man has had ischemic heart disease, high cholesterol, Type II diabetes…well that’s virtually every 70 year old man (JFG)*

*They are the really weird ones where you know that, realistically, are you going to bring them back? But at the same time, you don’t actually have a justification [for not discussing management options with the patient] other than that number of 95 or 90. (JFG)*

Common to both focus groups were descriptions of a lack of targeted communication that full resuscitation is not always in the best interests of the patient and that palliative care could be reframed as a form of “active care” or “doing everything that is appropriate.” It was perceived that, in some instances, in depth discussions about resuscitation modalities and escalation of care is clearly inappropriate, with a sensitive conversation about what will be done being more suitable.

*If someone’s going to die, if you’re not going to do CPR anyway, well then there’s no point mentioning all this. You may well say, “Look, they’re pretty unwell, if they continue to deteriorate, we’ll just keep them comfortable.” (CFG)*

Focus group participants agreed that a shared decision-making discussion with the patient should be used to reach a common understanding about the medical treatment plan. This may involve an interpretive approach, with the patient actively engaged in the decision-making when there are medical decisions to be made, and a deliberative (or directive) approach when there are no decisions to be made but information needs to be conveyed. Irrespective of the content of the discussion, participants agreed that the conversation needs to be open, honest and sensitive.

*It gives the patient the dignity and the power back to make a decision on what is important to them. (CFG)*

While the outcomes from the focus groups were generally consistent with the literature [19–22], concerns were identified in the literature that NFR orders can lead to inappropriately less intervention for potentially treatable causes of deterioration [21]. Consultants corroborated this with examples of patients who were documented NFR but who had been successfully resuscitated outside of the ward and of patients *“who should have been resuscitated and probably would have done well, but had been put for DNR inappropriately.”(CFG)*

#### Knowing how to say it

Gaps in training and mentoring of advance CPR decision-making communication were widely reported by participants. Concerns about junior doctors’ discomfort during CPR discussions and about causing the patient (or the substitute medical decision-maker) emotional distress were described, with junior participants feeling ill-equipped to address these concerns. The required skills are not explicitly taught in all medical schools and post-graduate training, and physicians reported variable exposure to mentoring opportunities—all considered important steps in becoming proficient in discussing end-of-life concerns. Participants recognised that communication skills can be learned, and should be taught and mentored. Junior doctors, however, were concerned with the inconsistent styles modelled to them by seniors.

Discussions about levels of care with all hospital admissions was suggested by a consultant as an option for ensuring that CPR-decision-making is part of standard care. Consultant focus group participants, more generally however, identified the need to broaden the dialogue about resuscitation by changing the focus from just CPR to viewing decision-making in the context of the overall treatment plan. Conversely, concern was expressed about expecting patients/relatives to make such big decisions and the potential for decision-makers to feel guilty about choices made on behalf of a family member. Both consultants and junior doctors proposed an alternative dialogue for cases where the escalation of care would clearly not benefit the patient. In such instances it was considered suitable to give people a realistic but sensitive explanation of the appropriate course of action.

*Maybe it’s not our role to put every big decision on the shoulders of relatives; they sometimes feel incredibly guilty about it. But I think we can’t be completely paternalistic, we need to at least have an appearance of respecting patient autonomy. (CFG)*

The literature review indicated that many barriers could be overcome by changing the focus to viewing CPR decision-making in the context of the overall treatment plan rather than a “tick box approach” [23]. Presenting such discussion as routine was identified as a way to ‘normalise’ potentially challenging conversations [19, 24]. Shared decision-making processes should include physicians interpreting the medicine within the patient context, and the patient and family conveying what is important to the patient [11, 25]. Involvement of patients during training can enhance physicians’ sensitivity to decision-making styles [26].

#### Wanting to say it

Participants identified the need for clinical leadership and engagement to counter inertia and lack of ownership of CPR decisions. Junior doctors expressed: moral distress when involved with resuscitation they felt inappropriate; anger and frustration at lack of ownership by consultants; and dread at being in charge of scenarios without any guidance. Junior doctors also reported concerns about feeling responsible for discussion about treatment escalation with patients with whom they were not familiar. Furthermore, consultants were concerned that juniors overestimated their abilities. While junior doctors reported that they could (and did) undertake these discussions successfully with guidance and consistent modeling, there was agreement that the responsibility for CPR decision-making rests with the consultant.

*What you start doing if you don’t call seniors with every single case is you start blurring the boundaries until you start making mistakes. (CFG)*

This being said, junior participants were concerned at the potential for litigation or complaint, particularly in the instance of CPR being conducted on someone who had a documented NFR order. However, lack of understanding by patients and family members of disease progression and the limitations of what CPR can do were perceived as barriers to holistic discussions with both patents and their families.

*They get really offended with us. You know, “but on TV they shock them and it sort of brings them back.” And you are like, “That’s TV. [It] doesn’t work that way.” (JFG)*

Time pressure was described by participants from both focus groups as an important barrier to pro-active planning for deterioration. While the discussion with the patient in itself was reportedly time-consuming, adequate preparation was also considered an essential component often not factored into the process.

Participants in the both focus groups consistently identified the expectation for ‘someone else’ to initiate advance care planning and that no one medical specialty owns goals of treatment decisions, which also has potential for ‘no-one to make the decision’. Both consultants and junior doctors reported that, in many cases, multiple opportunities to discuss treatment escalation with patients were ignored. Several participants suggested that advance care planning should take place in the primary care setting. Others considered discussions about treatment options to be the responsibility of the treating team, which should be undertaken within 24 h of an admission to hospital, rather than waiting until a deteriorating situation necessitated discussion. Participants from both focus groups, however, were concerned that discussions about treatment limitations were often undertaken at times of patient deterioration, by doctors who were not familiar with the patient, their values and priorities, or with the disease trajectory. The consultants acknowledged the uncertainty in predicting patient prognosis but highlighted the importance of shared decision-making and having a systematic approach to communication. To address these concerns, participants proposed a policy that a consultant is responsible for discussing care planning/escalation with all patients within 24 h of admission and documenting outcomes; whether the level of escalation is for all treatment intervention or for limited resuscitation.

The literature confirmed that patients and families are often unaware of the terminal status of the patient [27], but that health professionals underestimate patients’ willingness to discuss appropriate care [26, 28]. Research has previously demonstrated poor insight into substandard communication in end of life discussions with the tendency for doctors to perceive problems with other practitioners, not themselves [23].

### Development of the ‘two pronged’ intervention

#### Video resource to support staff education

Following the focus groups, a face-to-face meeting with the steering group and a film producer determined the characteristics of the video and the content to be included to overcome identified barriers to CPR decision-making (Table 2). Three sections were defined: i) clinical issues; ii) CPR decision-making framework; iii) communication tips and examples.

**Table 2:** Content of video ‘Advance CPR-decision-making in the hospital setting’

Section	Subsection	Time^a^	Content
A. The clinical issues (11:24)	1. The current situation	2:47	Frustration, CPR overuse, lack of decisions, variable approaches, poor communication^b^
	2. Why has this situation arisen?	5:09	CPR development, expectations, poor training, clinical uncertainty, ‘doing everything’^c^
	3. How can we improve clinical care?	3:29	Framework, normalize discussion, honesty, shared responsibility, scripted questions, involve team, systematize not protocolise
B. The decision -making framework (13:06)	1. Is CPR decision- making different?	3:09	Patient expectation, life and death, trust, part of overall care + ongoing
	2. The medical assessment	3:28	Answer ‘will this patient survive CPR’, how to make the decision
	3. Four clinical categories and discussion aim	4:38	Clinical framework presented in interview style, animation of framework, deliberate and interpretive communication [11]
	4. Documentation	1:59	Capture escalation plan, value + preferences of patient, follow local policy
C. Communication tips and examples (13:34)	1. Improving communication	5:13	Communication overview, clinician tips for CPR decision-making, learning communication, introduces tools ‘ask-tell-ask’ + ‘NURSE’
	2. Patient/Doctor scenarios	2:52	Poor conversation (tools annotated), Dot dies ‘bad death’, healthy view of death
	2.1 Dot and Dr Nick	5:29	Good conversation (tools annotated), Dot dies ‘good death’, consumer voice
	2.2 Dot and Dr Eng		
Overview video		5:35	Promotional style overview of Section A,B and C with dramatisation of dying scenes

^a^Minutes: seconds

^b^"Dot" clinical scenario introduced (would not survive CPR)

^c^Includes "Dot" and "Dr Nick" (without tools annotated), Dot arresting and rapid response teams commencing CPR, introduced animation of framework

The steering group guided script development and production of the video. An iterative consensus process was used to determine how to best approach advance CPR decision-making and how best to communicate and improve skills. The educational video aimed to capture the richness of clinicians’ views and experiences and the authenticity of the clinical setting. Critical feedback was sought from the peer review group regarding appropriateness of the video for education and patient safety, and additional information that should be included. Feedback guided further editing and additional filming to fill identified gaps (summarised in Additional file 1: Table S3). A second round of peer review elicited minor further feedback for video changes and recommended a facilitator’s guide be produced, which has been completed [29].

The resulting video included resuscitation scenes with a background emotive narrative illustrating two potential pathways towards death for a hospitalised patient. One pathway demonstrates poor communication leading to a death following CPR; the other, good communication leading to a ‘good death’ without inappropriate CPR.

The final video suite can be viewed online [29].

#### Goals of patient care form and process

The Victorian medical GOPC summary [14] was trialed in a ward setting. Audit data and local feedback guided evolution and modification of the GOPC summary and process. Audit demonstrated poor volume of documentation related to discussions and decision-making on the GOPC document and in the progress notes, with clinicians reporting difficulty locating earlier progress note entries. The Victorian medical GOPC summary was considered ‘too busy’, with excessive instructions and inadequate space for writing. The peer review group determined that the new, modified form should be clearer, with more free space and less reliance on progress notes. The peer reviewers recommended the term ‘goals of patient care’ be expanded to go beyond a medical treatment order but have the ability to act as an advance care plan with patient preferences and views documented on the same form. The peer review group proposed an expanded GOPC that could link the patient’s goals (preferences, values, expectations) to the medical goals (treatment intent and escalation plan) if clear documentation about the discussions and decision-making process were incorporated. The escalation plan was perceived to be more useful if, in addition to categorising the treatment goal, the escalation pathways and patient transfers within the health service were also defined (including use of ICU services). It was noted the appropriateness of ICU admission would need to be determined upon RRT clinical review. Consensus supported moving away from multiple tick boxes for individual treatments (such as use of inotropes or dialysis).

Many features of the Victorian GOPC summary/process worked well, including identifying surrogate decision-makers, receiving advance care planning, embedding CPR decisions within over-all medical treatment plans, the ability to endorse an order after hospital discharge and the four goal categories (ranging from Goal A: all appropriate life-sustaining treatment; to Goal D: end of life care: maintaining comfort & dignity). The “Terminal” category was expanded with the timeframe (current admission only) removed, noting patients may not die during the admission but palliation may be the ongoing treatment intent. Each category and its relation to the escalation systems is outlined in Additional file 1: Table S4, and can be adapted to local hospital settings. The changes aimed to reflect a balance between life prolonging treatment and symptomatic or palliative caring, and reflectcontemporary models of palliative care [30]. The modified GOPC form (Additional file 1: S5) has numerous potential advantages when implemented as part of routine clinical practice in contrast to existing NFR or components of care practice (Table 3).

**Table 3:** Comparison of features of a ‘components of care’ approach to NFR *versus* the ‘system of care’ approach captured in the Goals of Patient Care form in the hospital setting

‘Components of Care’ approach	‘Goals of patient care’ approach
Applies to small % of patients	Applies to large % of patients
Inconsistently records surrogate decision-maker and available advance care planning documents.	Routinely records surrogate decision-maker and availability of advance care planning documents.
Completed just before death, often by non-treating team	Proactively completed as part of routine care by treating team
	Part of overall treatment plan, with 4 specific goals
Medical escalation plan outlining use of individual components of treatment	Medical escalation plan which is goal and system oriented
Associated with sub-optimal care	Associated with improved quality of care
Misses patient preferences	Seeks and records patients’ goals, values and preferences.
Sequential model of care with sharp demarcation from life prolonging care to palliative care	Introduces symptomatic palliative care earlier in illness trajectory
Hospital, time limited, medical treatment order (ie doctor to doctor communication)	Orders can be endorsed beyond the current admission (potential to be an ongoing advance care plan)

## Discussion

Our study identified multiple barriers to advance CPR decision-making in the hospital setting, and include physician and system level barriers. We propose a two-pronged strategy to facilitate medical cultural change in the way CPR decisions and treatment limitations are made and documented in hospitals. The first relates to integration of educational messages based on consensus using videos as a teaching tool to address physician barriers. The second represents a process change, which is expansion of existing “goals of patient care” approaches [12, 14] to bring conversations, patient preferences and decision-making rationale together alongside the medical treatment goals. These changes aim to support clinical deterioration systems by assisting the recognition and response to patient preferences, and support improvements in end-of-life care. This approach may avoid excessive burdens and adverse effects from non-beneficial interventions [21].

Medical specialist training in clinical reasoning, ethics and communication is as important as teaching technical knowledge [19, 23, 28, 31–33]. The introduction of any new system for end-of-life decision-making should be accompanied by training and education, and include audit and follow-up processes [28]. Consistent with education theory, we used mixed media to ensure that complex skills were acquired and remembered. End-of-life decision-making skills may need to be repeated over years, demonstrated in clinical practice, then taught and mentored—resulting in incremental increases in professional skill levels [16].

The proactive GOPC approach supports “shared decision making” but also acknowledges that clinician or patient driven decisions may be appropriate in certain clinical scenarios. By using an ethical decision-making framework to explore the appropriateness of resuscitation and other treatments in the context of the patient’s overall medical condition/s, the likely outcomes of intervention and the patients’ preferences, we hope that useful information will be available for optimal ‘in the moment’ medical decisions during acute clinical deterioration. This often requires multiple conversations over time to build a picture of an individual’s values and priorities for care. Such conversations require clinical skill, and need to be personalised, account for life prolonging and palliative options, and be clearly documented and able to be applied between admissions.

Given that this was a pragmatic quality improvement project, elements of the intervention were developed and modified in response to peer review and feedback, rather than through a formal Delphi process. The project team’s objective was to promote in-advance CPR discussions and decision-making locally, thus, engagement of local and national experts and opinion leaders was considered a more appropriate process. However, formal evaluation of the interventions’ utility and safety in the clinical setting is required. Assessment of patient, family and health professionals’ responses to the intervention is also required.

## Conclusions

System-wide changes are needed to support decision-making, facilitate communication and handover of medical treatment plans between health professionals and across health settings. Our intervention aims to empower medical consultants and staff to drive system changes and build consensus in this complex area, with support from clinical leads. Health system and cultural changes, education and training, appropriate policy development and regular audit are important steps to normalise discussions and documentation of goals of care for all hospital patients. The ultimate measure of success will be alignment of treatment with the best interests of the patient.

Internationally, there is significant variability in advance CPR decision-making and implementation without a robust evidence-base for a clear way to improve care [34]. Through an iterative process of engagement and review, we have adapted a current decision–making model to improve patient care in this complex and problematic area.

## Additional file

Additional file 1: Table S1. Membership of the project Steering Group and Peer Review Group. **Table S2.** Barriers to routine advance CPR decision-making in hospitals with representative quotes. **Table S3.** Peer review group feedback about the content of the training video and corresponding modifications. **Table S4.** Categories and related escalation plan components of care on Goals of Patient Care Summary form. **S5.** Modified Goals of patient Care Form, front page. Modified Goals of patient Care Form, back page. (DOCX 690 kb)

## Abbreviations

CFG: Consultant focus group
CPR: Cardio-pulmonary resuscitation
GOPC: Goals of Patient Care
JFG: Junior focus group
NFR: Not-for-resuscitation
RRT: Rapid Response Teams
WA: Western Australia
