# Supplementary material for: Development of a video-based education and process change intervention to improve advance cardiopulmonary resuscitation decision-making
Source: BMC Health Serv Res. 2016 Oct 6;16:555. doi: 10.1186/s12913-016-1803-x (PMC5053041; doi:10.1186/s12913-016-1803-x)
Supplement: Additional file 1: Table S1. — Membership of the project Steering Group and Peer Review Group. Table S2. Barriers to routine advance CPR decision-making in hospitals with representative quotes. Table S3. Peer review group feedback about the content of the training video and corresponding modifications. Table S4. Categories and related escalation plan components of care on Goals of Patient Care Summary form. S5. Modified Goals of patient Care Form, front page. Modified Goals of patient Care Form, back page. (DOCX 690 kb) [file 12913_2016_1803_MOESM1_ESM.docx]

| Table S1. Membership of the project Steering Group and Peer Review Group | |
| --- | --- |
| **Discipline** | **n** |
| ***Steering Group*** | |
| Geriatric medicine | 2 |
| Intensive care | 2 |
| Palliative care | 2 |
| Emergency medicine | 1 |
| Junior doctors | 2 |
| Quality improvement | 1 |
| Health administrators | 2 |
| Academic medicine | 2 |
| ***Peer Review Group*** | |
| Geriatric medicine | 4 |
| Intensive care | 3 |
| Palliative care | 6 |
| Emergency medicine | 3 |
| Junior doctors | 5 |
| Quality improvement | 1 |
| Anaesthetics | 2 |
| Medical oncology | 1 |
| Postgraduate medicine | 1 |
| Department of Health | 6 |
| Paediatric palliative care | 1 |
|  | |

| Table S2. Barriers to routine advance CPR decision-making in hospitals with representative quotes | | | |
| --- | --- | --- | --- |
| **Themes with barrier (category)** | | **Focus group quotes** | |
| ***(i) Knowing what to say*** | | |  |
| Lack of knowledge (cognitive) | | Why don’t you bring up another scoring system for CPR? *(JFG)*  They [junior doctors] need to understand the differences between not for resus versus for active management and palliation. That still surprisingly confuses a lot of people (*CFG)*  And the registrar just wanted to treat all these things, despite discussing really clearly with the daughter and the daughter saying ‘I just want to make her comfortable *(CFG)* | |
| Lack of skill/expert clinical reasoning (cognitive) | | Prognostic uncertainty is there.*(CFG)*  And I think barriers for the junior staff in not discussing it are in a lack of understanding about the implications and also the different nuances of it as well, you know, completely not for CPR versus not for DC cardioversion, for intubation, for ICU, what are ceilings of care, are you still for active medical management, are you completely pulling out and palliating? *(CFG)*  Because you are not sure if your medical decision is feeding into the overall decision. *(JFG)* | |
| Lack of evidence utility (guideline) | | It’s quite difficult to actually define futile treatment. *(CFG)*  I’ve certainly seen stupid mistakes over the years in people who should have been resuscitated and probably would have done well, but they’ve been put for DNR inappropriately.*(CFG)* | |
| ***(ii) Knowing how to say it*** | | |  |
| Lack of self-efficacy (attitudinal) | | Maybe it’s not our role to put every big decision on the shoulders of relatives; they sometimes feel incredibly guilty about it. But I think we can’t be completely paternalistic, we need to at least have an appearance of respecting patient autonomy. *(CFG)*  It should be separated from them by you making the decision saying ‘we don’t think CPR would be beneficial’ … Seems to be more like ‘what do you think?’ *(JFG)*.  It’s more of a dialogue.*(JFG)*  I think by giving people a realistic expectation of what can happen, more often than not, you find they come to you and say, “I wouldn’t want that”*(CFG)* | |
| Lack of confidence in ability (emotive) | | The junior doctor came to them [the patient] on admission and was very shy and very uncomfortable just asking “do you want resuscitation?” *(CFG)*  At this point they may be very offended by raising it. *(JFG)*  They get really offended with us… ‘but on TV they shock them and it sort of brings them back’ and you are like ‘that’s TV. Doesn’t work that way’*. (JFG)* | |
| Lack of knowledge about patient (cognitive) | | I think it’s really harsh on poor ED staff. They’ve never met this family and they’ve been tasked with the idea of having to communicate a difficult conversation without having any prior understanding of where they are in their values or future health *(CFG)*  But it is nice if you some relationship with patient.*(JFG)* | |
| Lack of peer guidance and role models **(**physician) | | We need to give the junior doctors confidence with the education, with communication skills to be able to make this system work better. *(CFG)*  Given consultants have different ideas on what they expect makes it really hard. *(JFG)*  I find you can learn a lot from seeing a good consultant do it. *(JFG)* | |
| Conflicting culture (patient) | | Patients are not aware of the outcome [of CPR]; they are probably overestimating that they’re going to be saved. *(CFG)*  It took almost 2 hours for us to explain to the whole family and then the outcome was good, but it took almost 3 days for us to [get to that point]. It totally depends upon next of kin if they are not able to make a decision, it is hard for treating doctors.*(JFG)*  Some of that seems to be guilt as well. They don’t want to say ‘oh just let mum die.’ *(JFG)* | |
| ***(iii) Wanting to say it*** | | |  |
| Lack of accurate self-assessment (attitudinal) | Now she’d been in hospital a number of days, she must have been seen by multiple doctors, at probably multiple levels….but not a single person has considered limitations of escalation of therapy. *(CFG)* You hope that conversation had already been had by the GP or the treating team. *(JFG)*  What you start doing if you don’t call seniors with every single case is you start blurring the boundaries until you start making mistakes.*(CFG)*  [Junior registrars] think they know a vast amount more than they really do.*(CFG)* | | |
| Lack of motivation (physician) | I do [wards] where literally the interns have conversations about CPR and all of the limitations of treatment because the Rego and consultants were not interested *(JFG)* | | |
| Legal concerns (physician) | Most we follow criteria like setting the situation for even all those things but even then sometimes we might face some troubles from the family.*(JFG)* | | |
| Time and support (resource) | Sometimes you’ve got to speak with the patient and family and you can’t spend half an hour with every single patient (CFG)  You think you are going to need a lot of time to set aside for this discussion and in a quiet place*.(JFG)* | | |
| Workload/overload (system) | I think there’s time pressures, and there’s also perceived lack of priority.… it takes time to do it properly *.(CFG)* | | |
| Organizational (process) | …their progression to resuscitation should be charted and should be considered within 24 hours. *(CFG)* | | |
| JFG = Junior focus group  CFG = Consultant Focus Group | | |  |

| Table S3. Peer review group feedback about the content of the training video and corresponding modifications | |
| --- | --- |
| **Feedback/Discipline of respondent** | **Modifications to video** |
| ***Appropriateness of content?*** |  |
| Consider avoiding term “futility” as is contentious *(PC)* | Grabs with futility removed from section A. One grab in video B remains. |
| The “preference misdiagnosis” grab doesn’t fit in. *(Anaesthetist, Registrar)* | Removed section |
| ***Video style*** |  |
| CPR scenes are excellent and very authentic*(ICU, PC)*  Some of advanced life support protocol is incorrect (*ICU)*  There is not enough urgency in CPR scene, doctors smiling is inappropriate. *(PC)* | Use of CPR scenes extended throughout video to break sections. Amendments made with minor errors kept in (but discussed in the facilitator’s guide) |
| Having too much data may detract *(Registrar)* | Data slides simplified in section 2a for CPR survival, and for 4 categories. |
| Useful to show that doctors are part of a team and that different members can provide useful input into end-of-life decision-making *(policy-maker)* | Additional filming of NW with team inserted in section A. Additional footage of team meeting, nurse discussing with patient, family members present in section C |
| ***Video content*** |  |
| “uncertainty is a key concept – needs to come up earlier *(Policy-maker)* | Uncertainty grab moved to section a1 and also inserted in 2c |
| Intensive care consultants quote about “doing nothing” is a wrong message – from my perspective we never do nothing *(PC)* | PS^ quote inserted “we always do everything” |
| We are trained to save lives but that is not always under our control. *(PC)* | Inserted PS quote “dying is not failure its only failure if this is not identified or managed” |
| Clearly situate the discussion about CPR in its broader context of discussing goals of care and specific treatment limitation choices*(PC)* | Inserted PS quote emphasizing that CPR decision is in context of overall patient care; grab stating the importance of clarifying goals so unwanted treatment not provided |
| - No conversation is perfect but that if we are trusted and seem to care and address issues with humanity, our imperfect words are forgiven. *(PC)*  - Good conversation uses the term “fighter” and no support person*(PC)*  - Bad conversation – some good aspects such as siting down and introducing himself *(PC)* | Moved away from titles of good and bad discussion – instead illustrating use of Tools in both dialogues.  Voice over emphasizes to examine conversation in context of group learning and analyse separately. |
| The bit where you mention the tools (SPIKES^#^, NURSE*^ǂ^* etc) is too fast (*ICU)* | Grab removed and changed to voice over only including “ask-tell-ask” and NURSE tools. |
| Need statement emphasizing that the key issue is ownership and denial of the problem by medical staff (*ICU)* | insert “the problem is us” quote |
| Communication section missing clinical hints on starting the conversation with open questions (*ICU)* | Add ‘You must have seen a lot of people in the last few days, tell me in your own word what is going on” |
| Take home message about communication and how to improve missing *(PC)* | Add “communication is like two wings of a bird” |
| ***Use in education / practice*** | |
| Video- shouldn’t be used in isolation to generate discussion *(Academic)* Useful for medical students and GP’s as well as hospital doctors (*Anaesthetist)* | Facilitators guide subsequently developed to support blended learning teaching strategies. |
| We are using SPICT + Surprise question together and this is capturing everyone we should be offering a conversation about CPR with. (*PC)* | Section B2 voice over included surprise question* and filming of SPICT tool˚ |
| I do a lot of communication skills teaching, and at present I only use brief (3 minutes or so) clips to give people a break from role plays! (*ICU)* | Videos broken into sections – with communication section provided separately. |
| ^PS: Peter Saul *Surprise question: Would you be surprised if this patient died within the next 12 months? ˚SPICT tool: Supportive and Palliative Care Indicators Tool, *Highet G, et al. BMJ Supportive & Palliative Care 2013;0:1–6. doi:10.1136/bmjspcare-2013-000488 ^#^* SPIKES: A Six-Step Protocol for Delivering Bad News: *Baile W, et al. The Oncologist 2000, doi:10.1634/theoncologist.5-4-302. ^ǂ^*NURSE: Patient-centred Interviewing: An Evidence-based Method *Smith RC, Lippincott Williams & Wilkins. 2002.*  Smith RC. Patient-centered interviewing: an  evidence-based method. Philadelphia: Lippincott  Williams & Wilkins; 2002.  Smith RC. Patient-centered interviewing: an  evidence-based method. Philadelphia: Lippincott  Williams & Wilkins; 2002. | |

| Table S4. Categories and related escalation plan components of care on Goals of Patient Care Summary form | | | | |
| --- | --- | --- | --- | --- |
| **Category** | **A** | **B** | **C** | **D** |
| **Treatment intent** | **Cure focus – the default position** | **Cure focus with limitations** | **Non-burdensome treatment with symptom control** | **Palliative care focus** |
| * |  | | | |
| CPR | **+** | **-** | **-** | **-** |
| Intubation | **+** | **+/-** | **-** | **-** |
| ICU admission | **+** | **+** | **-** | **-** |
| MET call ** | **+** | **+** | **+/-** | **-** |
| ICU outreach # | **+** | **+** | **+/-** | **-** |
| Transfer off ward | **+** | **+** | **-** | **-** |
| Symptom focus | **-** | **-** | **+/-** | **+** |
| Terminal Phase | **-** | **-** | **-** | **+/-** |
| * Black with white dots =Life prolonging care; White with black dots in the triangle=Symptomatic palliative care; White with black dots in the rectangle=Full palliative care including terminal care  *  *  ** All patients can have staff or family initiated MET calls if worried or require assistance for patient care (e.g. acute distress)  # ICU outreach is a consultative service which reviews patients prior to MET call criteria, where staff are worried or teams wish to have second opinion. | | | | |

S5 Modified Goals of patient Care Form, front page

A5 Modified Goals of patient Care Form, back page
